# First person – Hagen Gegner

**DOI:** 10.1242/bio.049783

**Published:** 2019-12-16

## Abstract

First Person is a series of interviews with the first authors of a selection of papers published in Biology Open, helping early-career researchers promote themselves alongside their papers. Hagen Gegner is first author on ‘High levels of floridoside at high salinity link osmoadaptation with bleaching susceptibility in the cnidarian-algal endosymbiosis’, published in BiO. Hagen conducted the research described in this article while a PhD Candidate in Christian R Voolstra's lab at King Abdullah University of Science and Technology (KAUST), Thuwal, Saudi Arabia. He is now interested in symbiotic interactions in the marine environment by combining physiological measurements with metabolomics, and is currently working as an editorial professional at Frontiers, Switzerland.


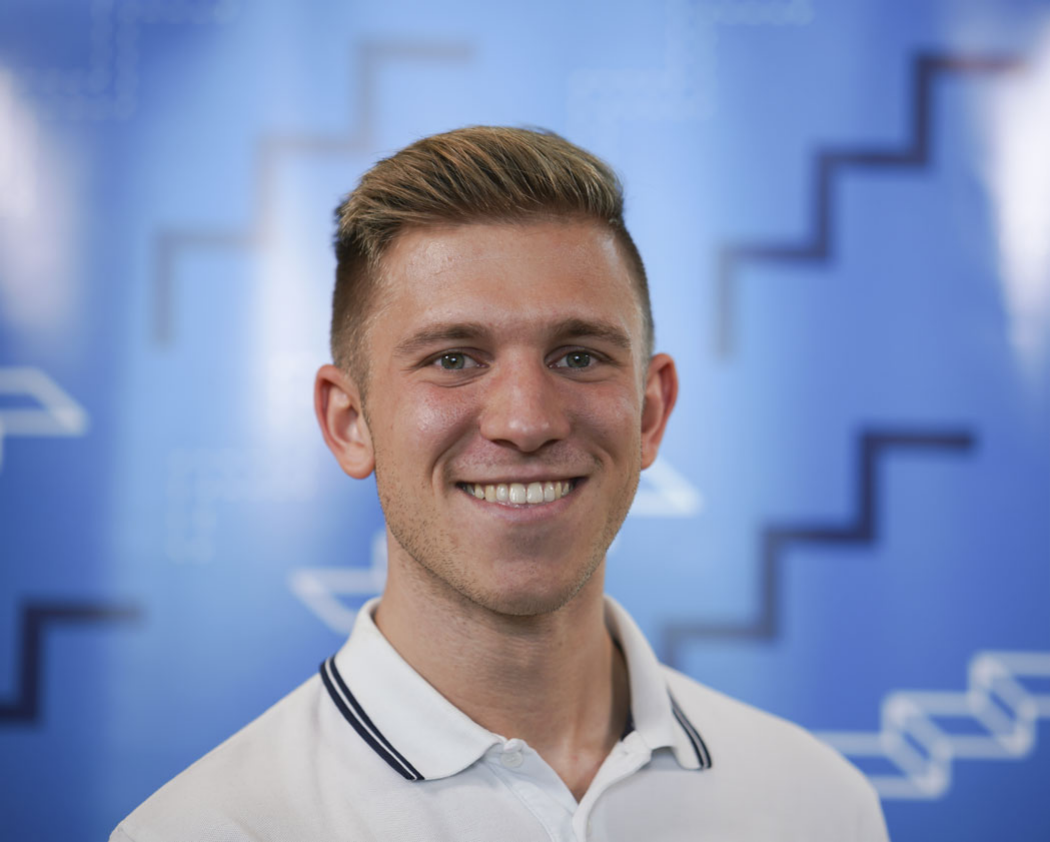


**Hagen Gegner**

**What is your scientific background and the general focus of your lab?**

I am a marine molecular biologist with a background in protein expression and fish population genetics who is now getting more and more into metabolomics.

The lab focuses on all aspects of the coral holobiont (coral host, its algal partners and the microbiome) using a multi-omics approach.

**How would you explain the main findings of your paper to non-scientific family and friends?**

Coral bleaching, the loss of the photosynthetic algal partner from the coral tissue, is happening globally due to ocean warming. However, not all corals bleach at the same temperature, which raises the question: what affects the differences in bleaching susceptibility of corals? For example, corals from the Red Sea tolerate extremely high temperatures during the summer months, making Red Sea corals well known for their bleaching resistance. Since the Red Sea is an extremely saline basin, we started wondering if this bleaching resistance of corals from the Red Sea is connected to the salinity they are exposed to. Our previous findings, also published in BiO (Gegner et al., 2017), indicate that there is indeed a connection between high salinity and bleaching resistance in Aiptasia, a tiny anemone that we use as a coral model organism.

In our current study, we picked up where we left in 2017, focusing on the mechanism behind this link of high salinity and bleaching resistance. For that, we looked at the molecules produced during bleaching in low, medium and high salinity in Aiptasia associated with two different algal partners. In doing so, we identified one molecule called floridoside, a sugar and major player during osmoadaptation (the adaptation to changes in the salinity of the sea water) in corals. Floridoside increased in concentration with higher salinity in one of the two Aiptasia combinations that also showed a higher bleaching resistance. Intriguingly, floridoside, besides being an osmolyte, also acts as an antioxidant. Antioxidants protect cells by detoxifying them of reactive oxygen species (ROS). If the level of ROS is too high, it causes damage to the cells, the DNA inside, as well as other cell components. In symbiotic corals and anemones, high levels of ROS that damage the photosystem and disrupt symbiosis are a hallmark of bleaching. Besides the higher levels of floridoside that aligned with a higher bleaching resistance in high salinity, we also measured lower levels of ROS in the algal partners, indicating that floridoside may be mitigating the negative effects of heat on the symbiosis in high salinity. Therefore, we propose that osmoadaptation is linked to the bleaching susceptibility in anemones and corals by osmolytes such as floridoside that can mitigate bleaching.

**What are the potential implications of these results for your field of research?**

Due to the rapid decline of corals globally, it is crucial to understand the mechanisms behind coral bleaching. While we understand the result of ocean warming on corals (or other factors that induce bleaching), the mechanisms behind bleaching and vice versa, of bleaching resistance, remain elusive. Our findings highlight the importance of the environment, e.g. salinity, as an overlooked environmental factor that is a crucial component in determining the bleaching resistance of symbiotic cnidarians, like corals. These findings may help to understand the reported heightened bleaching resistance of corals from highly saline environments such as the Northern Red Sea or the Persian Arabian Gulf.

“Due to the rapid decline of corals globally, it is crucial to understand the mechanisms behind coral bleaching.”

**What has surprised you the most while conducting your research?**

Although more research on salinity has been conducted since our first BiO publication on that topic in 2017, salinity as an environmental factor is still underrepresented in most marine studies, such as coral research. As such, it surprises me that not more people are including salinity in their measurements, as it is clearly a factor that influences the metabolism of all invertebrates, such as corals (including all organisms in this symbiosis) since they are ‘osmoconformers’. While being an osmoconformer implies a passive strategy, it certainly requires tremendous cellular metabolic changes of the organism. Just as a cherry hanging from a cherry tree would pop during heavy rain due to immense osmotic stress, osmoconformers must adjust their osmolytes continuously to avoid a similar fate. Portraying this in the case of the bleaching susceptibility of cnidarians, I hope to raise awareness on the importance of environmental conditions for future studies.

**Figure:**
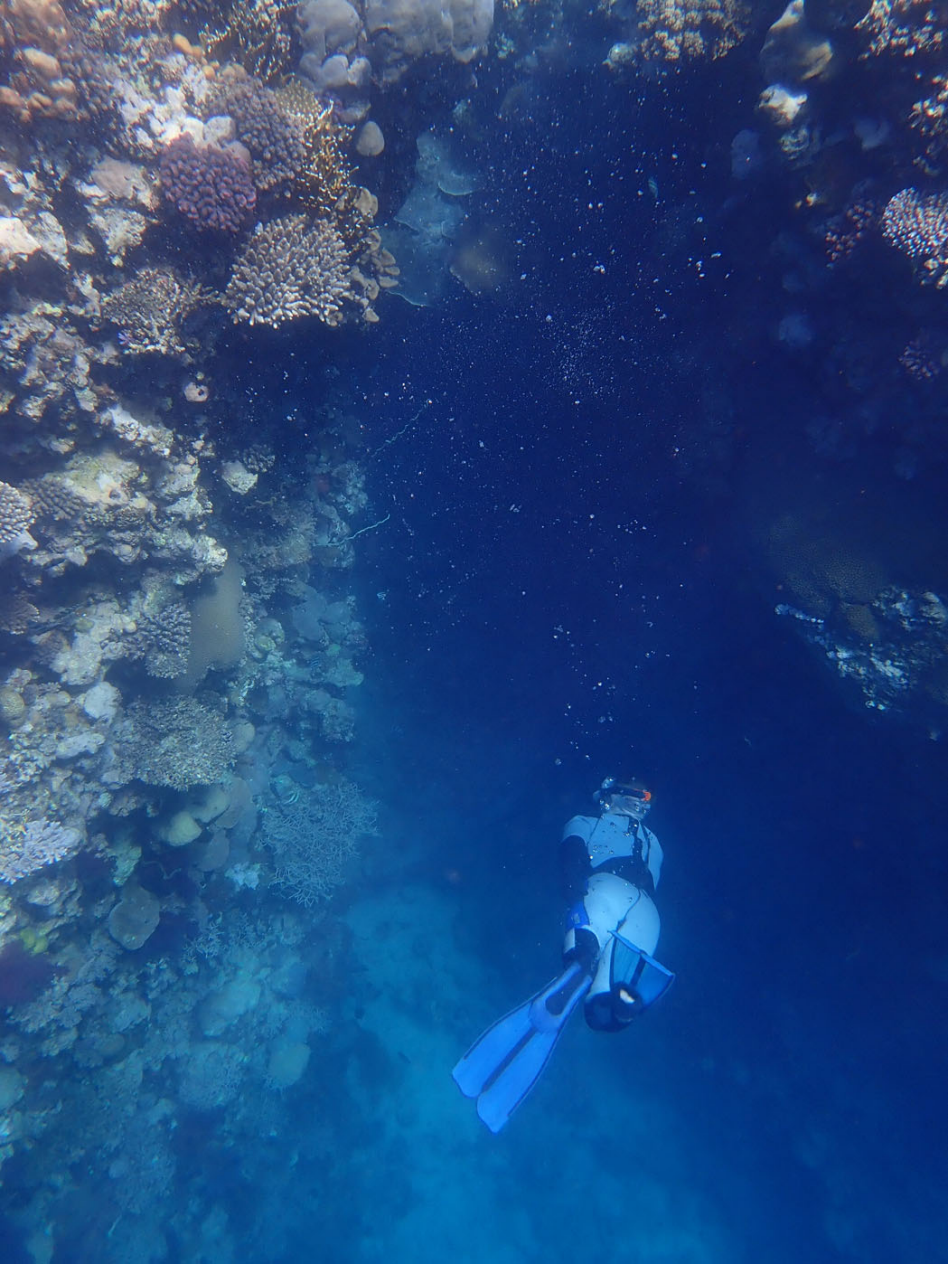
**The Red Sea coast of Saudi Arabia close to the Red Sea Research Center at KAUST provides stunning reefs and great opportunities for coral research.** Corals can be reached easily from the shore or sampled via a short boat trip from the university′s wet lab facilities. I took full advantage of that, enjoying the snorkelling and diving during work as well as in my free time!

**What, in your opinion, are some of the greatest achievements in your field and how has this influenced your research?**

One of the greatest achievements in my field is the use of model organisms to better understand coral symbiosis. For over 60 years coral research has been focused on corals. The field was dominated by reef studies in remote areas or aquarium experiments in the same regions. Yet, corals are hard to get and even harder to maintain, which hinders the number of scientists who can conduct studies on corals. With the onset of coral model organisms, researchers all over the world, including myself, can attempt to unravel this symbiosis in a systematic and controlled manner. Additionally, it opens the entire field for more functional studies to better understand mechanistically what happens during bleaching in corals. This will lead to more research, which in turn will result in more findings and a better understanding of corals. Ultimately, I am hopeful that this approach will increase the chances of helping corals threatened by climate change.

“Ultimately, I am hopeful that this approach will increase the chances of helping corals threatened by climate change.”

**What changes do you think could improve the professional lives of early-career scientists?**

In my opinion, we, as scientists, must move away from misleading career-defining measures such as H-Indices and Impact Factors that are frequently used in academia. These metrics are hindering collaboration and facilitating research that is focused on impact rather than the science itself. This bias, in the long run, creates false-positive findings that waste funding and time. On top of that, it also builds barriers for early-career scientists in excelling in their fields and to find a ‘normal’ work-life balance. To counter this, the entire scientific community should avoid these metrics, embrace international as well as interdisciplinary collaborations and Open Access knowledge.

**What's next for you?**

I aim to further help with disentangling symbiotic interactions in the coral holobiont and hope to be involved in other marine-related science. While this is my overall goal, I have yet to determine from which position I best try to achieve that, being it in the editorial or academic world.
